# Special theme on HIV and disability - time for closer bonds

**DOI:** 10.1186/1758-2652-12-26

**Published:** 2009-11-09

**Authors:** Shirin Heidari, Susan Kippax

**Affiliations:** 1 International AIDS Society, Geneva, Switzerland; 2 National Centre in HIV Social Research, University of New South Wales, Sydney, Australia

## Abstract

With the success of antiretrovirals and increased access to this lifesaving treatment, the life expectancy of people living with HIV has been substantially increased and, in many instances, is comparable to that of the general population. However, HIV infection, as well as its treatment, can cause physical, psychological or social disabilities that prevent people living with HIV from full and equal participation in society. At the same time, there is evidence that people with disabilities are at greater risk of contracting HIV. Although more attention is being paid to these overlapping fields, the field of HIV and disability remains largely overlooked.

The *Journal of the International AIDS Society *is publishing, for the first time, a thematic section consisting of a number of papers on HIV and disability to provide readers with an update of developments in the field.

## Editorial

Disabilities associated with HIV infection are of great concern. Physical, mental or sensory impairment caused by the HIV infection or its treatment can potentially interact with the functional status of people living with HIV, making it difficult for them to participate fully and equally in society.

At the same time, there is evidence that people with disabilities are at greater risk of contracting HIV infection. People living with disabilities are often stigmatized and marginalized and, in many cases, as a result of associated discrimination and violence, are unable to access HIV prevention and health education appropriate to their needs. Furthermore, for some, the discrimination they suffer is exacerbated by accompanying issues of poverty and gender inequality.

According to the United Nations special unit on the Rights and Dignities of Persons Living with Disabilities, more than 650 million people worldwide are living with mental, physical, social or medical disabilities; 80% of them are residing in developing countries, and many of these countries have a high burden of HIV [1].

A key step towards recognizing the rights of people with disabilities is the Convention on the Rights of Persons with Disabilities and its Optional Protocol that came into force on 3 May 2008 [2]. The convention recognizes and reaffirms the human rights of people living with any kind of disabilities. As a legally binding human rights instrument, the convention obliges nation states to fully protect the human rights of people living with disabilities and to ensure reasonable accommodation for people living with disabilities so that they are able to fully exercise and enjoy all human rights on an equal basis with others [2]. Non-discrimination and full participation, as well as accessibility and equal opportunity, are among the guiding principles of this convention [2].

Research on HIV and disability - with regard to both the disabling consequences of HIV infection and its treatment, and the vulnerability to HIV of people living with disability - has been sorely neglected. Despite the dismaying lack of adequate efforts in this area, some positive developments have been made. As an example, at the latest International AIDS Conference, held in Mexico City in August 2008, several sessions in the programme were dedicated to this important topic, where the need for more focused efforts was recognized. This topic will also be addressed at the International AIDS Conference in Vienna, Austria, in July 2010; its theme is *Rights Here, Right Now*.

Through this special issue, the *Journal of the International AIDS Society *would like to raise awareness on issues of HIV and disability. Readers are encouraged to comment on the individual articles by posting their comments on the journal website. Submission of additional research articles or commentaries on this topic is also warmly welcomed.
